# Motor Experts Care about Consistency and Are Reluctant to Change Motor Outcome

**DOI:** 10.1371/journal.pone.0161798

**Published:** 2016-08-30

**Authors:** Volker Kast, Christian Leukel

**Affiliations:** 1 Department of Sport Science, University of Freiburg, Germany; 2 Bernstein Centre Freiburg, University of Freiburg, Germany; Waseda University, JAPAN

## Abstract

Thousands of hours of physical practice substantially change the way movements are performed. The mechanisms underlying altered behavior in highly-trained individuals are so far little understood. We studied experts (handballers) and untrained individuals (novices) in visuomotor adaptation of free throws, where subjects had to adapt their throwing direction to a visual displacement induced by prismatic glasses. Before visual displacement, experts expressed lower variability of motor errors than novices. Experts adapted and de-adapted slower, and also forgot the adaptation slower than novices. The variability during baseline was correlated with the learning rate during adaptation. Subjects adapted faster when variability was higher. Our results indicate that experts produced higher consistency of motor outcome. They were still susceptible to the sensory feedback informing about motor error, but made smaller adjustments than novices. The findings of our study relate to previous investigations emphasizing the importance of action exploration, expressed in terms of outcome variability, to facilitate learning.

## Introduction

Humans can quickly modify movements they once acquired. This ability can be investigated using an experimental paradigm called visuomotor adaptation [1]. Here, visual input is suddenly displaced by e.g. prismatic glasses while executing a motor task [2]. The subjects are required to adapt to this displacement, and mechanisms driving adaptation have been frequently evaluated based on the subjects’ behavior.

The most popular mechanistic explanation is that visuomotor adaptations are learned via sensory prediction errors [3]. This means that the brain predicts the sensory outcome of the movement, compares this prediction with actual sensory feedback, and subsequently corrects for the error between the two by modifying the corresponding motor command. Importantly, learning through sensory prediction errors does not depend on task success. Success does not mean that a predefined goal was reached but is rather achieved when the error between sensory prediction and actual sensory feedback is zero. To exemplify this point, a handball player that is aiming for the right side of the goal but hits the center will generate a sensory prediction error despite the fact that the throw will be successful. In contrast to learning with sensory prediction errors, recent studies proposed a learning process that is sensitive to task success [4, 5]. This process simply reinforces behavior that proved to be successful. A previous study aimed to promote reinforcement when subjects reached performance asymptote during adaption. Subjects were informed about task success with an auditory cue (termed binary error (BE) feedback), which indicated whether a predefined target was hit. Notable, BE consisted of solely auditory information but no visual feedback. As the main result, subjects receiving BE showed slower forgetting of the adapted movement than subjects receiving full visual feedback containing information about task success plus vector error feedback (VE+BE) [5].

In a recent study [6] we compared visuomotor adaptation between experienced handball players (experts) who mastered the tested movement, a standardized free throw, and untrained subjects (novices). The experts adapted at a slower rate than the novices, and they also forgot the adaptation slower. Considering the result of Shmuelof et al. (2012), this latter finding led us to hypothesize that experts naturally utilize learning processes that depend on task success to a greater extent than novices. In the present study, we continued to test experts and novices and used the same experimental setup as Shmuelof et al. (2012). Based on our speculations about learning processes in experts, we initially hypothesized that all experts, including those who receive visual feedback (VE+BE), would naturally utilize processes that depend on task success to a larger extent than novices. Thus there would be no additional benefit from BE in terms of slower forgetting in experts. As a result, the slower forgetting in subjects with BE, as observed by Shmuelof et al. [5], would also be seen in our novices receiving BE, but not in experts.

Indeed, the present data show this result. However, more detailed analyses did no longer support our assumption. The data rather indicate that the way experts responded to task success was in principle not different compared to novices, but experts were more reluctant in modifying motor output. This was beneficial in terms of outcome variability and persistency, but increased the time required to adapt to the visual displacement, and likely also to forget the visual displacement.

## Materials and Methods

We tested 37 healthy right-handed subjects (aged 23 ± 4 years, 16 females, 21 males), who were divided into two groups and matched with respect to age, height, weight and gender. Different inclusion criteria existed for each of the two groups. Novices (18 in total, 8 females, 10 males) had not regularly performed ball sports (particularly volleyball, basketball, handball) and other throwing sports (e.g. javelin throw), as members of a club or in their free time. Experts (19 in total, 9 females, 10 males) were amateur or semi-professional handball players (German 3rd Bundesliga or Landesliga) with at least 12 years of experience in competitive club handball. All participants were unaware of the purpose of the study. They did not previously participate in similar experiments, had normal or corrected to normal vision, and gave written informed consent for participation. The study was approved by the local ethics committee of the University of Freiburg (161/15) and performed in accordance with the Declaration of Helsinki (latest revision in Fortaleza, Brazil, 2013).

### General Experimental Setup

**Fig 1:**
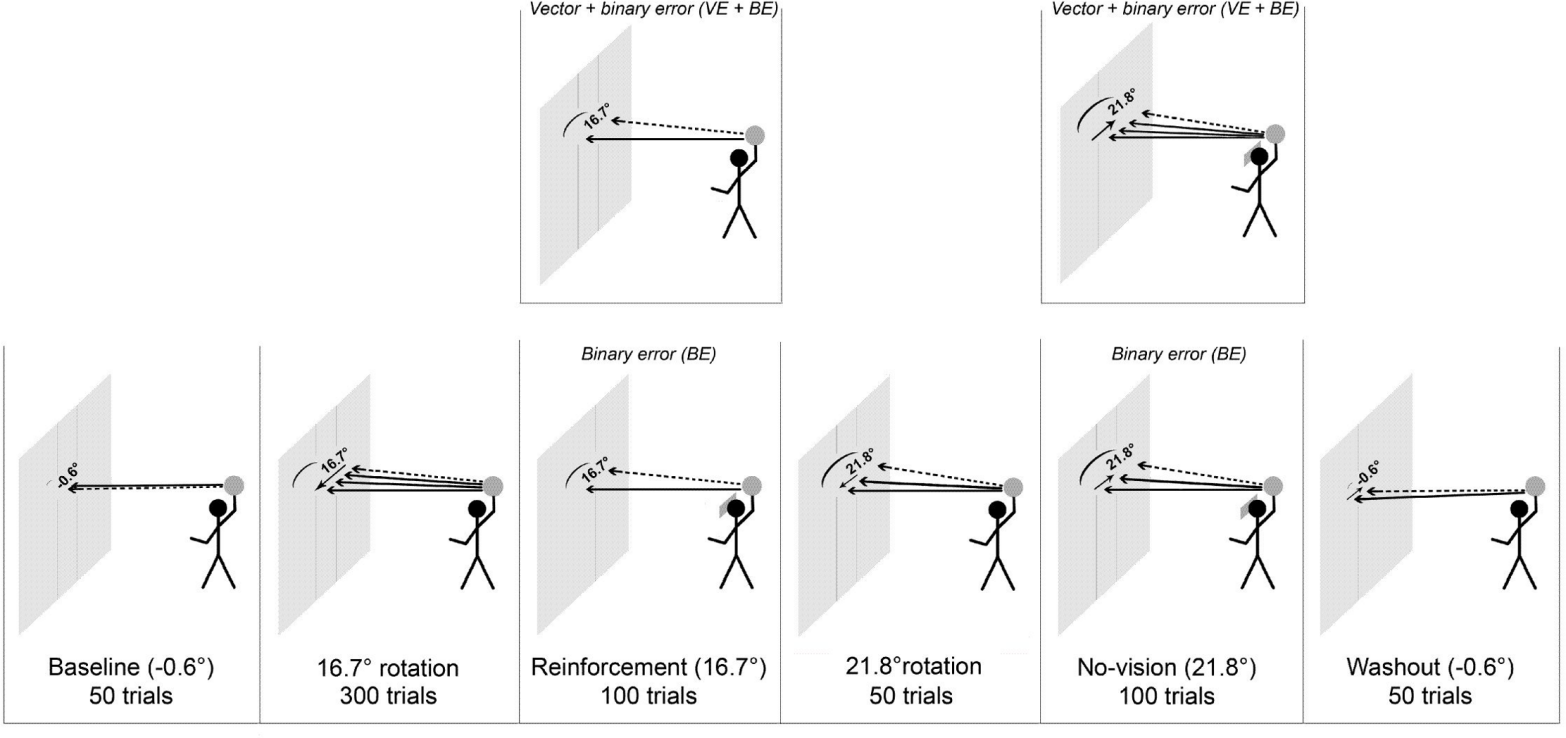
illustrates the experimental procedure. The tested motor task was a visuomotor adaptation of handball free throws with prismatic glasses. Handball players (experts) and subjects without experience in ball sports (novices) were equally divided into two groups, a group receiving visual vector error feedback and binary error feedback (VE+BE), and a group receiving binary error feedback (BE), in a distinct phase of the experiment. The reduction to BE was made possible by shutter glasses obstructing vision. The visual shift induced by the prismatic glasses is indicated with dotted arrows for every phase of the experiment. The resultant throwing directions throughout the phase are displayed with the solid arrows. Starting with a series of baseline throws, all subjects first adapted to 16.7° visual displacement. Thereafter, this adaptation was reinforced in subjects with BE through auditory feedback about task success (binary error, BE). Subjects with VE+BE received full visual feedback about vector error (VE) and task success (BE) in this phase of the experiment. Adaptation to additional visual displacement of 5.1° (to a total displacement of 21.8°) followed for all subjects, prior to the no-vision phase and washout of the adaptation.

All subjects participated in a single session. There were two experimental protocols, randomly and equally distributed within both groups. The protocols differed with respect to the applied visual feedback in a specific phase of the experiment (see Fig 1). One half of the subjects (experts: 9 subjects; novices: 9 subjects) received normal visual feedback in this specific phase, containing information about task success plus vector error feedback (VE+BE), whereas the other half (experts: 10 subjects; novices: 9 subjects) was only provided with auditory feedback about task success (BE, analogue to the study introducing this experimental protocol [5]). The reduction to BE was made possible by shutter glasses (PLATO visual occlusion spectacles, Translucent Technologies®, Toronto, Canada) obstructing vision.

The tested motor task was a visuomotor adaptation of handball free throws with prismatic glasses [2]. The participants stood with their left foot behind a blue line at a distance of 3.5 m to a wall. The right foot stood laterally behind the left one. Subjects threw with a soft ball (foam ball with 20 cm diameter) to a wooden panel attached to the wall. The panel had the approximate dimensions of a handball goal (2.80 m wide, 2.10 m high). Subjects were asked to throw the ball at a target (30 cm wide), which was bounded with two vertical tape stripes on the panel. We drew additional black vertical lines (interspaced by 10 cm) to the left and to the right side of the target to assess horizontal direction errors. We only cared about horizontal but not vertical directional errors, and vertical errors were not recorded in the present study. Subjects wore prismatic glasses with different angles of deviation and additionally the shutter glasses on top of them during the different phases of the experiment (Fig 1). Throwing was performed with the right arm. The arm was moved behind and over the trunk before the ball left the hand. It was important that subjects did not see the ball until it had left their hand. Such unwanted visual feedback could enable participants to orient the arm in line with the direction of the target and therefore bias the effect of the prismatic glasses. To prevent this bias, the shutter glasses had a 15 cm long blinder on the right side. In 10 test throws without prismatic glasses before the start of the experiment, subjects were asked if they saw the ball or the arm while throwing despite the blinder. This was never the case.

### Experimental Procedures

All participants performed 50 baseline throws, while wearing sham prismatic glasses with a visual displacement of -0.6° (minus indicates that the displacement was leftwards) (Fig 1). The sham glasses were introduced to keep experimental conditions similar, so that subjects always looked through prismatic glasses. This aimed to minimize e.g. the effect of contextual cues affecting performance.

Next, subjects were exposed to a 16.7° visual rotation, causing a rightward visual shift of 105 cm (30 cm per meter) at the panel. Changing prismatic glasses occurred while the subjects closed their eyes, and they were instructed to open their eyes again after the experimenter had completed the change. The subjects performed a series of 300 throws (approximate duration: 20 to 25 min), for which they received full visual error feedback. The experimenter repeated the sentence “throw where you are seeing the target” after every twenty shots in this adaptation phase and also in all of the consecutive phases of the experiment. This instruction aimed to prevent the subjects from applying cognitive strategies for throwing.

In the subsequent reinforcement phase of 100 throws immediately executed after adaptation, the 16.7° visual rotation was maintained (Fig 1), and subjects with VE+BE kept getting full visual error feedback while subject with BE received only binary auditory feedback from one of the experimenters (“Yes” in case of a hit, “No” in case of a miss). A “Yes” indicated that the ball hit the panel between the two vertical tape stripes (target width: 30 cm). Visual feedback in subjects with BE was controlled via the shutter glasses, manually triggered by an experimenter, occluding vision when the ball was leaving the hand. The solution to use manual triggering of the shutter glasses worked perfect in our setting, subjects never saw the ball and their right hand. The shutter glasses were opened again when handing the ball back to the subjects, prior to starting a new trial. It is important to note that the ball was handed at the right side of the body, and subjects were not allowed to turn their head and look at the ball. After every twenty throws, the subjects received full visual feedback in one trial, similar to the study of Shmuelof et al. [5]. These full vision trials ensured that the subjects could again orientate themselves in case of a long period of “No” trials.

Hereafter, the protocol for all subjects (receiving BE and VE+BE) was the same again for all remaining phases. Prismatic glasses were changed from 16.7° to 21.8° for the following 50 throws (see Fig 1) to prompt further adaptation [5]. The 21.8° caused a rightward visual shift of 140 cm (40 cm per meter) at the panel.

In order to examine motor forgetting, the consecutive no-vision phase (100 throws) was crucial. Participants kept the prismatic glasses of 21.8°, and threw the ball without visual feedback, which was taken away with the aid of the shutter glasses in the same way as in the previous reinforcement phase, as described before. Binary auditory feedback was not provided in this phase.

Finally, in the remaining 50 trials of the experiment, subjects changed again to the sham prismatic glasses (-0.6°) and received full visual feedback for washout of the adaptation. There was no longer pause between the phases, only the short breaks in which the prismatic glasses were changed. None of the subjects, even untrained novices, complained about fatigue during or after the experiment. Thus the total number of 550 throwing movements in our experiment were not exhausting.

### Data Analysis and Statistics

Horizontal directional errors of each trial were detected in steps of 5 cm by visual inspection from one experimenter as in our previous experiment [6]. The vertical lines on the panel were displaced by 10 cm, and the minimal step of 5 cm results from the criterion whether the ball hit a line or hit in between two lines. The experimenter assessing the directional errors was always the same person throughout all conducted experiments. We made the experience that the assessment of horizontal directional errors by visual inspection is highly reliable [6]. After the measurement, these values were calculated as degree rates. The throws that hit the right side of the panel with respect to the midline are indicated as positive values, and the throws that hit the left side are indicated as negative values.

To prepare the data for statistical comparison, horizontal directional errors were binned in bins of 10 trials (one BIN represents the mean of 10 trials) in each subject. Data were analyzed using parametrical tests, i.e. analyses of variance (ANOVAs), Student’s T-tests, and bivariate correlation analyses. Greenhouse-Geisser corrected values for ANOVAs are reported in case sphericity of the tested samples was violated. Statistics were performed using SPSS 23 (IBM®, Armonk, NY, USA). Mean and standard error of the mean (SEM) are reported.

## Results

### Baseline Phase

**Fig 2:**
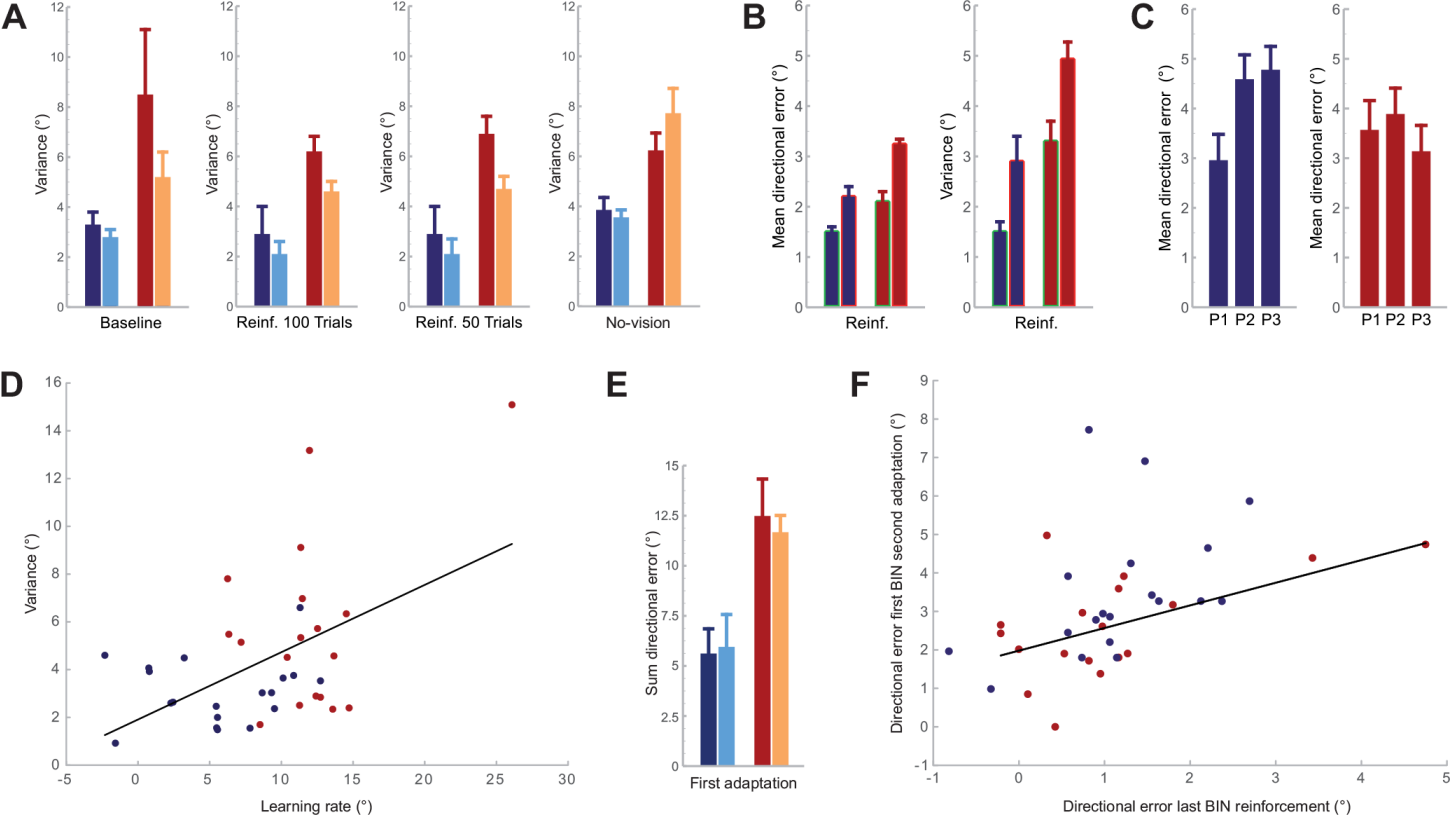
**A** displays the variance (group mean and standard error of the mean, SEM) calculated in the different phases of the experiment, the baseline phase, the reinforcement phase (100 trials and first 50 trials), and the no-vision phase. The colors label the different groups and subgroups: experts with BE (dark blue), experts receiving VE+BE (light blue), novices with BE (dark red), and novices receiving VE+BE (yellow). **B** shows changes of the horizontal directional error between successful trials and the corresponding subsequent trials (framed in green), and the same for non-successful trials (framed in red), for subgroups receiving BE in the reinforcement phase (blue: experts; red: novices). The individual mean and the individual variance of the changes was calculated. The bars refer to the group mean values (for mean and for variance). Bars indicate SEM of the group mean values. **C** shows the directional error (group mean and SEM) for distinct parts in the no-vision phase. We divided the 100 trials of the no-vision phase in three parts (33 trials each), termed P1, P2, and P3, and calculated the individual mean values for each of the 33 trials. The blue bars in this graph refer to the experts and the red bars refer to the novices. **D** shows individual subject data of two variables, learning rate in the adaptation phase (summed changes in directional error of the first 20 trials) and variance in the baseline phase, plotted against each other. The blue dots refer to the experts and the red dots refer to the novices. The black line depicts the linear regression of all data. **E** shows the learning rate in the adaptation phase (group mean and SEM) for the tested groups and subgroups separately. **F** displays the directional error of the last BIN of the reinforcement phase and the directional error of the first BIN of the second adaptation phase for each subject. Blue dots refer to the experts and the red dots refer to the novices. The black line shows the linear regression of all data.

In our previous study, experts showed less variability of directional errors than novices in this phase [6]. We therefore computed variability (variance) of directional errors of all throws in the baseline phase for each subject in the present experiment. This variable was compared between groups (experts and novices) and subgroups (BE and VE+BE). The ANOVA showed a significant effect for GROUP (F_1,33_ = 6.9, P = 0.01), but no effect for SUBGROUP (F_1,33_ = 1.7, P = 0.2), and no effect for GROUP x SUBGROUP (F_1,33_ = 0.84, P = 0.37). These results indicate that variability of movement errors was lower in experts than in novices (Fig 2A).

### First Adaptation Phase

**Fig 3:**
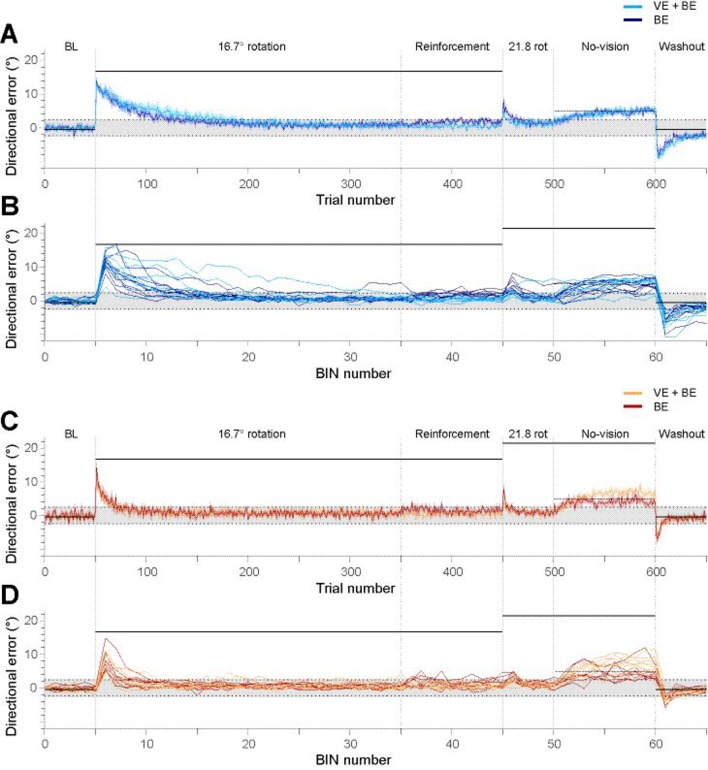
Displays horizontal directional errors of throws made throughout the experiment. Parts A and B display data of experts, C and D display data of novices. Parts A and C display group mean values (solid lines) and SEM (shaded areas) of single trials. Parts B and D shows single subject data (in BINS of trials). The colors refer to the different subgroups: experts with BE (dark blue), experts receiving VE+BE (light blue), novices with BE (dark red), and novices receiving VE+BE (yellow). Horizontal solid black lines show visual displacement induced by the prismatic glasses. The dashed line in the no-vision phase shows the earlier rewarded location, and the dotted lines at -2.45° and 2.45° indicate the boundaries of the target (shaded area) that had a width of 30 cm. Note that directional errors within these boundaries were rated as success in the reinforcement phase of the experiment.

We tested for differences in directional errors between groups and subgroups and therefore entered the values of 30 BINS (300 trials) into an ANOVA. There was a significant effect for BIN (F_3.7,115_ = 81, P < 0.001), a significant effect for GROUP (F_1,33_ = 11.6, P < 0.01), a significant effect for BIN x GROUP (F_3.7,115_ = 13.6, P < 0.001), no significant effect for SUBGROUP (F_1,33_ = 0.62, P = 0.44), no effect for BIN x SUBGROUP (F_3.7,115_ = 0.76, P = 0.54), and no effect for BIN x GROUP x SUBGROUP (F_3.7,115_ = 0.86, P = 0.49). These results indicate that experts adapted slower than novices (Fig 3).

Experts in this study produced less variability of errors during baseline throws and adapted slower than novices. A recent study showed a significant correlation between variability of movement error at baseline and the rate of learning in subsequent adaptation [7]. We therefore performed the same bivariate correlation analysis (including all subjects) as Wu and colleagues [7] between the variability of horizontal directional errors during the baseline phase and changes in directional errors during adaptation. The learning rate in some previous studies was expressed by fitting exponential or power functions to the data. However, we [6] and others [5] made the experience that fitting works only for a fraction of the learning curves. In many others, coefficient of determination is too low to validly represent the data. We therefore decided not to fit the data but to calculate the sum of the differences of the horizontal directional errors of the first 20 trials in each subject

$$\sum_{t=1}^{19}\left(e_{t}-e_{t+1}\right)$$

where t is the first trial and e is the horizontal directional error of the respective trial. Our analysis showed a significant correlation (r = 0.50, P < 0.01), and this finding indicates that the rate of changes subjects produced during adaptation was related to the variability of the directional errors in the baseline phase (Fig 2D and 2E). Although there are significant behavioral changes within the first 20 trials, one may argue that this number is too low to validly reflect the learning rate. We therefore calculated additional bivariate correlation analyses between the variability of directional errors and the sum of the differences of horizontal directional errors of the first 10, 30, 40 or 50 trials, respectively, of the adaptation phase. The result is the same for all analyses, namely a significant correlation between variability and learning rate (10 trials: r = 0.45, P < 0.01; 30 trials: r = 0.4, P < 0.05; 40 trials: r = 0.39, P < 0.05; 50 trials: r = 0.35, P < 0.05). This indicates that the number of learning trials included into the bivariate correlation analysis did not importantly affect the statistical outcome. A further result we would like to mention with respect to the analysis of the learning rate is that its size did not relate to the size of the directional error of the first trial in the adaptation phase. A bivariate correlation analysis of the learning rate (calculated based on the first 20 trials) and the horizontal directional error of the first trial showed no significant correlation (r = 0.13, P = 0.44).

Before the reinforcement phase, all subjects were supposed to have adapted to the visual displacement by the prismatic glasses, without differences between groups and subgroups. To test this, we analyzed the horizontal directional error at the very end of the adaptation phase, for the final 10 trials, between groups and subgroups with an ANOVA. Indeed, we found no differences between groups and subgroups. The ANOVA showed no effect for TRIAL (F_9,297_ = 0.76, P = 0.65), no effect for GROUP (F_1,33_ = 0.27, P = 0.61), no effect for SUBGROUP (F_1,33_ = 0.01, P = 0.93), no effect for TRIAL x GROUP (F_9,297_ = 0.64, P = 0.76), no effect for TRIAL x SUBGROUP (F_9,297_ = 1.1, P = 0.35), and no effect for TRIAL x GROUP x SUBGROUP (F_9,297_ = 1, P = 0.41) (Fig 3).

### Reinforcement Phase

Half of the subjects in this phase received binary error feedback (BE), i.e. only verbal information about task success, while the other half received full visual feedback containing vector error feedback and binary error feedback (VE+BE). We tested for differences in horizontal directional errors between groups and subgroups and therefore analyzed values of the 10 BINS (100 trials) of this phase. There was a significant effect for SUBGROUP (F_1,33_ = 31, P < 0.001), but no effect for BIN (F_5.3,176_ = 0.29, P = 0.98), no effect for GROUP (F_1,33_ = 0.29, P = 0.59), no effect for BIN x GROUP (F_5.3,176_ = 1.57, P = 0.17), no effect for BIN x SUBGROUP (F_5.3,176_ = 0.84, P = 0.53), and no effect for BIN x GROUP x SUBGROUP (F_5.3,176_ = 1.46, P = 0.2). These results confirm the visual impression in Fig 3 that subjects with BE showed larger directional errors than subjects with VE+BE. In fact, the reduced BE feedback may likely have changed the success of movements compared to subjects with full visual feedback. We therefore tested whether the success rate during the reinforcement phase was different between groups and subgroups. Note that a horizontal error below +2.45° and above -2.45° was rated as success, which indicates that the ball hit inside the target width of 30 cm framed by the vertical black lines (see Fig 1). The ANOVA showed an effect for GROUP (F_1,33_ = 16.3, P < 0.001) and also for SUBGROUP (F_1,33_ = 22.7, P < 0.001), but no effect for GROUP x SUBGROUP (F_1,33_ = 2.7, P = 0.11). The respective values were: Experts: BE: 74 ± 3% (group mean ± standard error of the mean, SEM); VE+BE: 93 ± 1%; Novices: BE: 67 ± 4%; VE + BE: 76 ± 3%. These results indicate that success rate was higher in experts than in novices and that subjects with VE+BE showed higher success rates than subjects with BE.

In most of the 100 trials of the reinforcement phase, subjects with BE received only verbal information about task success. However, in 4% of the trials, subjects received full visual feedback, for orientation. The trials with visual feedback could have caused stronger movement corrections, resulting in larger changes of directional error compared to trials with BE feedback. To analyze this aspect, in subjects with BE we calculated the difference D in horizontal directional errors between the trial where they received visual feedback and the trial thereafter

$$D={|e}_{t}-e_{t+1}|,$$

for the 20^th^, 40^th^, 60^th^ and 80^th^ trial. We contrasted these values to the difference in horizontal directional errors in trials with just BE feedback, for the 10^th^, 30^th^, 50^th^, and 70^th^ trial. The sum of these errors were compared between BE experts and BE novices with an ANOVA. There was no effect for FEEDBACK (BE versus full visual feedback) (F_1,17_ = 2.4, P = 0.14), no effect for GROUP (F_1,17_ = 3.6, P = 0.08), and no effect for FEEDBACK x GROUP (F_1,17_ = 1.1, P = 0.31). Thus trials with full visual feedback did not result in larger movement corrections than trials in which BE was provided. The corresponding values were: Experts: BE: 7.2 ± 1.1° (group mean ± SEM); full visual: 7.8 ± 0.6°; Novices: BE: 8.1 ± 1.3°; full visual: 11.1 ± 1.3°.

A recently conducted study suggests that, when actions are reinforced in a similar way as in the present study, reward causes a smaller change of the outcome in the subsequent trial than a lack of reward [8]. We therefore calculated changes of the horizontal directional error between successful BE trials and the corresponding subsequent trials (absolute values), and the same for all non-successful BE trials. The individual mean and the individual variance of this analysis, separately for successful BE trials, and non-successful BE trials, was calculated. Note that from the 100 trials being analyzed, in novices 66.8 ± 4 trials were successful, and 33.2 ± 4 trials were non-successful. In experts, 74.3 ± 2.8 trials were successful, and 25.7 ± 2.8 trials were non-successful. The values (mean and variance) were analyzed with an ANOVA with respect to the factors SUCCESS (successful and non-successful trials) and GROUP (experts receiving BE and novices receiving BE). The ANOVA for the mean values showed an effect for SUCCESS (F_1,17_ = 68, P < 0.001), an effect for GROUP (F_1,17_ = 24, P < 0.001), but no effect for SUCCESS x GROUP (F_1,17_ = 3.51, P = 0.08). The ANOVA for the variance values showed an effect for SUCCESS (F_1,17_ = 22, P < 0.001), an effect for GROUP (F_1,17_ = 16.4, P < 0.01), but no effect for SUCCESS x GROUP (F_1,17_ = 0.014, P = 0.91). Further, based on the individual mean values, we calculated the relative increase in movement error in non-successful trials in relation to successful trials. Non-successful trials led to a 146 ± 9% (group mean ± SEM) increase in movement error compared to successful trials in experts. In novices, the corresponding value was 160 ± 12%. This relative increase was not significantly different between experts and novices (unpaired Student’s T-test: P = 0.39). Altogether, these results indicate that the change in horizontal directional errors was higher for non-successful trials than for successful trials, and that experts made smaller changes than novices. Importantly, however, the relative changes in movement error between successful trials and non-successful-trials was not different between experts and novices (Fig 2B).

As variability was smaller in the baseline phase in experts, we tested whether variability during the reinforcement phase was also different between groups and subgroups and analyzed both the full 100 trials and the first 50 trials of the reinforcement phase with an ANOVA. We analyzed the first 50 trials separately as we suggested, based on results obtained by Shmuelof et al. [5], that BE causes higher variability especially in the initial part of the reinforcement phase. For 100 trials, the ANOVA showed a significant effect for GROUP (F_1,33_ = 45, P < 0.001) and for SUBGROUP (F_1,33_ = 8.1, P < 0.001), but no effect for GROUP x SUBGROUP (F_1,33_ = 0.87, P = 0.36). For 50 trials, the ANOVA showed a significant effect for GROUP (F_1,33_ = 41, P < 0.001) and also for SUBGROUP (F_1,33_ = 9.1, P < 0.01), but no effect for GROUP x SUBGROUP (F_1,33_ = 1.8, P = 0.19). These results indicate that experts in general produced less variability of directional errors than novices, and that variability of directional errors were larger for the BE groups than for the VE+BE groups (Fig 2A).

### Second Adaptation Phase

We were interested in whether horizontal directional errors were different between groups and subgroups and therefore analyzed 5 BINS (50 trials) with an ANOVA. There was a significant effect for BIN (F_2.9,97_ = 55, P < 0.001), an effect for BIN x SUBGROUP (F_2.9,97_ = 9.3, P < 0.001), but no effect for GROUP (F_1,33_ = 2.5, P = 0.12), no effect for SUBGROUP (F_1,33_ = 1.3, P = 0.27), no effect for BIN x GROUP (F_2.9,97_ = 0.8, P = 0.50) and no effect for BIN x GROUP x SUBGROUP (F_2.9,97_ = 0.98, P = 0.41). These results indicate that for some BINS, BE groups showed higher values than VE+BE groups. The data displayed in Fig 3 suggests a larger magnitude of directional errors for BE groups than for VE+BE groups in the initial part in the second adaptation phase. To test whether this was the case, we analyzed the directional error of the very first BIN separately by means of an ANOVA, and performed the same analysis also for the fifth BIN. The ANOVA for the first BIN showed a significant effect for SUBGROUP (F_1,33_ = 14.1, P < 0.001), an effect for GROUP (F_1,33_ = 4.6, P < 0.05), but no effect for GROUP x SUBGROUP (F_1,33_ = 0.74, P = 0.40). The ANOVA for the fifth BIN showed no effect for GROUP (F_1,33_ = 1.62, P = 0.21), no effect for SUBGROUP (F_1,33_ = 0.11, P = 0.74) and no effect for GROUP x SUBGROUP (F_1,33_ = 0.01, P = 0.92). These results confirm that the size of the horizontal error of the initial trials of the second adaptation phase was different between subgroups (Fig 3). The data displayed in Fig 3 not only shows higher directional errors in the initial part of the second adaptation phase for subjects with BE, but also higher values than subjects with VE+BE at the end of the adaptation phase. We therefore asked whether the size of the horizontal directional error at the beginning of the second adaptation phase was related to the size of the horizontal directional error produced at the end of the reinforcement phase. We performed a bivariate correlation analysis between the last BIN of the reinforcement phase and the first BIN of the second adaptation phase. The correlation was significant, for the BE groups (r: 0.5, P < 0.01), and also when including all subject data (BE and VE+BE subgroups) into the analysis (r: 0.42, P < 0.01). These findings indicate that a higher horizontal directional error at the end of the reinforcement phase led to a higher horizontal directional error in the second adaptation phase, not only in subjects with BE feedback but in all tested subjects (Fig 2F).

### No-Vision Phase

Vision in this phase was occluded with shutter glasses when the ball left the hand, and thus the subjects received no information about the movement error. When signals informing about movement error are withheld, performance gradually declines back to baseline values observed at the beginning of the adaptation. In subjects that were naïve to the executed motor task, BE feedback in contrast to full visual feedback in the reinforcement phase resulted in a slower forgetting in the subsequent error-clamp phase [5]. We therefore assessed whether directional errors were different between groups and subgroups in the no-vision phase of our experiment (10 BINS; 100 trials). The ANOVA showed an effect for BIN (F_5.7,187_ = 30, P < 0.001), a significant effect for BIN x SUBGROUP (F_5.7,187_ = 2.9, P < 0.05), a significant effect for BIN x GROUP x SUBGROUP (F_5.7,187_ = 3.9, P < 0.001), but no effect for GROUP (F_1,33_ = 1.21, P = 0.28), no effect for SUBGROUP (F_1,33_ = 2.7, P = 0.11), and no effect for BIN x GROUP (F_5.7,187_ = 1.5, P = 0.18). These results indicate that forgetting at some BINS was larger in subjects with VE+BE than in subjects with BE, and, most importantly, that the difference in forgetting was significantly greater between the novices’ BE and VE+BE groups than between the experts’ BE and VE+BE groups (Fig 3). At first sight (Fig 3), it may appear that the novices’ BE group behaved similar to the experts’ BE and VE+BE group. However, when taking a closer look, in the initial part of the phase the novices appeared to forget in a similar way than both expert groups, but then stabilized their performance in contrast to the experts. The experts seem to slowly and continuously drop towards baseline performance. Thus forgetting may be different between the novices BE group and the experts BE group. To examine this, we separated the 100 trials of the no-vision phase in three parts (33 trials each), and calculated the individual mean of the horizontal directional errors for each of the parts. We thought that three parts best capture the modulation of errors seen in Fig 3. Mean values of the three parts were analyzed between groups (BE novices and BE experts). The ANOVA showed an effect for PART OF PHASE (mean of first, second, and third 33 trials, respectively) (F_2,34_ = 12.4, P < 0.001), an effect for PART OF PHASE x GROUP (F_2,34_ = 15.6, P < 0.001), but no effect for GROUP (F_1,17_ = 0.6, P = 0.45). These results strengthen the visual impression that experts and novices with BE show different patterns of changes. While forgetting in novices stayed at a similar level until the end of the no-vision phase, experts continuously decayed at a slow rate (Fig 2C).

We finally tested whether the variability of horizontal directional errors was different between groups and subgroups. The ANOVA showed a significant effect for GROUP (F_1,33_ = 23.2, P < 0.001), no effect for SUBGROUP (F_1,33_ = 0.67, P = 0.42), and no effect for GROUP x SUBGROUP (F_1,33_ = 1.7, P = 0.2). This finding indicates that variability of horizontal directional errors was smaller in experts than in novices (Fig 2A).

### Washout Phase

We compared horizontal directional errors between groups and subgroups (5 BINS). The ANOVA showed a significant effect for BIN (F_2.6,85_ = 76, P < 0.001), a significant effect for GROUP (F_1,33_ = 28, P < 0.001), a significant effect for BIN x GROUP (F_2.6,85_ = 4, P < 0.05), no effect for SUBGROUP (F_1,33_ = 0.57, P = 0.46), no effect for BIN x SUBGROUP (F_2.6,85_ = 1.2, P = 0.3) and no effect for BIN x GROUP x SUBGROUP (F_2.6,85_ = 0.86, P = 0.45). These results indicate that experts de-adapted slower than novices. This result replicates our earlier findings [6] (Fig 3).

## Discussion

From the very first trials in our experiment, a key marker indicating differences between experts and novices was the variability of the outcome of the throws. Variability was always lower in experts than in novices. Motor variability has often been ascribed to the physiological noise of the sensory and motor system [9], causing unwanted uncertainty of motor output [10]. While it is true that physiological noise may produce motor variability, variability per se is sometimes not unwanted but rather welcome. This especially refers to learning. For instance, juvenile songbirds were found to express larger vocal variability than adults [11], which was considered to be a key promoter of learning [12]. In the human motor domain, the idea that variability is relevant for learning was tested in a recent experiment. Wu et al. [7] found that the amount of variability expressed during baseline behavior predicted the learning rate in reinforcement learning and also in sensorimotor adaptation. The lower the outcome variability in the baseline trials the slower the subjects learned the subsequent task. Higher variability was argued to allow for better exploration of the unknown and thus facilitate learning, by sacrificing accurate performance to some extent. Rather than being a static resultant of physiological properties, Wu and colleagues [7] showed that the amount of variability was actively regulated by the motor system in context of the environment in which learning took place, and that this regulation could be persistent and remembered from one day to the next. In our study, we found a correlation between variability of baseline performance and the learning rate, like in the study of Wu et al. [7]. There was a marked difference in variability and learning rate between experts and novices. This clearly separated these two groups. Whether this difference in variability between the two groups was also actively steered by the motor system, to promote learning in novices and guarantee task success in experts, or a feature resulting from the optimization of corresponding neural sensory and motor circuitries in experts producing physiological noise, is an interesting but unresolved question.

The slow change of motor output found in the experts does not necessarily mean that they ignored the sensory feedback containing the error. We rather assume that they essentially treated the error similar than the novices but were reluctant in adjusting motor output. Evidence for this assumption can be found in the reinforcement phase of our experiment. In this phase, subjects with BE feedback received solely auditory information, but no visual feedback. They were verbally informed after every throw by the experimenter about whether the ball hit the target (indicating a successful movement) or did not hit the target (indicating a non-successful movement). A previous study providing BE feedback in a similar manner to the present study indicated that, after a movement was successful, the relative change in motor outcome in the subsequent trial was reduced compared to when the movement was non-successful [8]. The study of Pekny et al. [8] tested healthy subjects displaying the results we just described, and also patients with Parkinson’s disease. Parkinsonian patients showed the same modulation of motor outcome after successful movements than healthy subjects, but a smaller modulation after non-successful movements. This result, displaying an altered modulation compared to healthy individuals for non-successful trials only, was interpreted as an impaired ability to increase variability in case of non-successful trials based on altered brain circuits in the patients. In our study, changes in motor outcome after successful and non-successful trials in experts and novices receiving BE feedback was also analyzed. But our findings did not yield a single-sided shift like in the patients tested by Pekny and colleagues [8]. In fact, we observed that the magnitude of change for both, non-successful trials and successful trials, was lower in experts than in novices. This finding supports our assumption made at the beginning of this paragraph, that the experts were indeed sensitive to sensory feedback informing about error, but that smaller changes were made than in novices.

Besides the lower variability and decreased learning rate, the slower forgetting of motor outcome was a prominent feature of the experts’ behavior. Slower forgetting in experts was apparent in the washout-phase, and this result replicates findings of our earlier study [6]. We further argue that slower forgetting was also seen in the no-vision phase of the current experiment. In this phase, directional errors in experts with BE and VE+BE gradually and steadily changed towards baseline performance, and we expect that this change would have continued in case more trials would have been measured.

In contrast to the behavior of the experts during the no-vision phase stands the behavior of the novices. Novices with BE feedback forgot to a certain extent and then appeared to keep this change until the end of the no-vision phase. This result, that subjects kept the change, was similarly found by Shmuelof et al. [5], albeit this study used error-clamp trials in contrast to no-vision trials performed by our subjects. The replicability is interesting in itself, as Shmuelof et al. [5] investigated small arm movements and subjects in our study performed a gross motor task. Shmuelof and colleagues [5] ascribed the slower forgetting to the reinforcement of actions, which was in their view promoted by BE feedback. However, instead of emphasizing BE feedback, one could also emphasize the visual feedback that was provided in 4% of the trials in the reinforcement phase. In our experiment this was necessary, indicated by pilot tests without visual feedback, as especially some of the novices would likely have started to drift heavily outside the target zone without further visual guidance. With respect to these 4% of the trials, it has long been known that intermittent visual feedback improves retention of the learned movement [13]. An important difference between previous studies applying intermittent feedback [13] and the setup of Shmuelof et al. [5] and also the setup of the present study, however, is that in the latter two cases the trials in between the visual feedback trials contained binary information. This significant difference makes direct comparisons of the results and speculations about underlying mechanisms impossible. In fact, we do not have a satisfying answer as to why novices with BE showed this intriguing behavior, namely a steady outcome better than baseline performance after initial forgetting, in the no-vision phase. We, however, may speculate that it was indeed reinforcement learning that caused this stability of performance. Outcome variability, a predictor of the learning rate, was higher in novices with BE than in novices with VE+BE in the reinforcement phase. A bigger exploration by higher variability may not only allow for faster learning but also better memorization of what was learned. Decay may thus depend on the stability of the motor memory [14]. In this sense, a better memorization based on BE feedback in novices may be the reason for the stability of motor outcome in the no-vision phase. This interpretation would mean that the slower forgetting seen in novices with BE and experts with BE and VE+BE can be likely ascribed to different mechanisms. In experts, slower forgetting would be related to a reluctance to change, whereas in novices it would be based on better learning.

A puzzling issue we would like to address is why the error size at the very beginning of the adaptation phase was smaller than the visual displacement induced by the prismatic glasses (16.7°). Ideally, subjects should exactly display this deviation in terms of the horizontal directional error at least in the first throw during adaptation. In fact, most of the subjects showed errors around this value, but some subjects displayed much lower values, which lowered the average error displayed in Fig 2. We can only speculate about why this was the case. In novices, perhaps a reason could be related to the high variability of their performance, while in experts a lower error than expected could be related to the reluctance to a change of motor output. Independent of the reason, we do not think that the smaller initial error biased the results and related interpretations, as the phenomenon occurred equally in both groups and in both subgroups.

In summary, this study showed that variability of motor outcome was lower in motor experts than in novices, and that the variability was positively correlated with the learning rate during motor adaptation. We believe that the smaller learning rate in experts does not mean that they were insensitive to sensory feedback. Rather, we believe that the smaller learning rate reflects the reluctance to change motor output, which may also be responsible for a slower change of motor outcome in the no-vision and the de-adaptation phase.

We may conclude with a linkage to practical sports, as coaches often complain that movements in highly-trained athletes can hardly be modified. A possible solution for successful modification, derived from the present results, may be to increase movement variability by training interventions, enhancing again their learning ability.

## Supporting Information

S1 Dataset (CSV)

S2 Dataset (CSV)
