# Correction: Motor Experts Care about Consistency and Are Reluctant to Change Motor Outcome

**DOI:** 10.1371/journal.pone.0165855

**Published:** 2016-10-27

**Authors:** The PLOS ONE Staff

Fig 3 has been corrected for increased readability. The publisher apologizes for the error.

**Fig 3:**
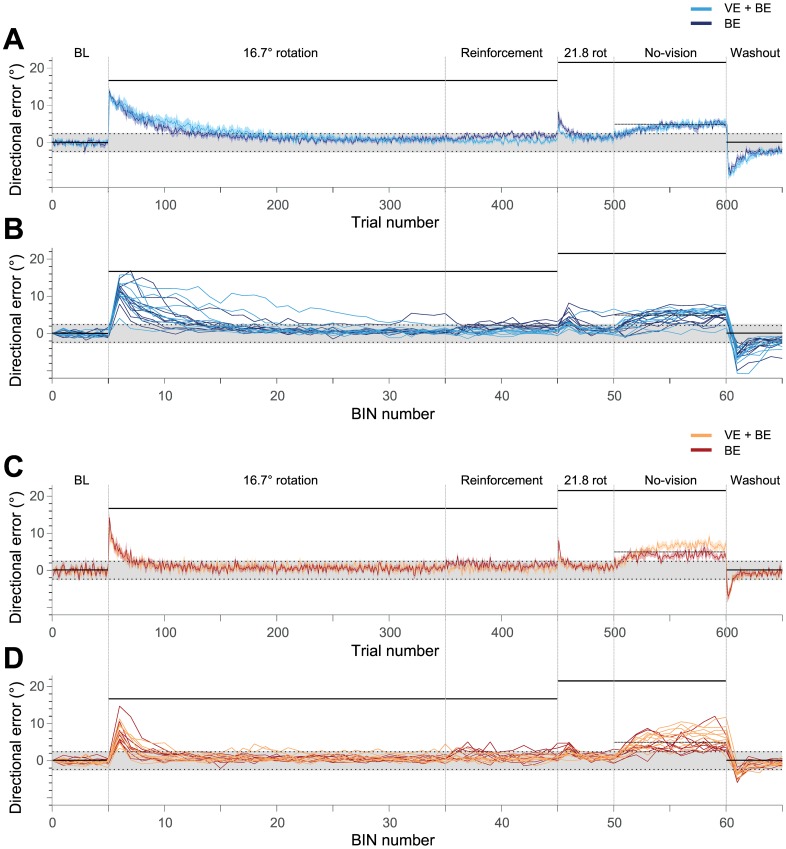
Displays horizontal directional errors of throws made throughout the experiment. Parts A and B display data of experts, C and D display data of novices. Parts A and C display group mean values (solid lines) and SEM (shaded areas) of single trials. Parts B and D shows single subject data (in BINS of trials). The colors refer to the different subgroups: experts with BE (dark blue), experts receiving VE+BE (light blue), novices with BE (dark red), and novices receiving VE+BE (yellow). Horizontal solid black lines show visual displacement induced by the prismatic glasses. The dashed line in the no-vision phase shows the earlier rewarded location, and the dotted lines at -2.45° and 2.45° indicate the boundaries of the target (shaded area) that had a width of 30 cm. Note that directional errors within these boundaries were rated as success in the reinforcement phase of the experiment.
